# Methylation of Breast Cancer Predisposition Genes in Early-Onset Breast Cancer: Australian Breast Cancer Family Registry

**DOI:** 10.1371/journal.pone.0165436

**Published:** 2016-11-30

**Authors:** Cameron M. Scott, JiHoon Eric Joo, Neil O’Callaghan, Daniel D. Buchanan, Mark Clendenning, Graham G. Giles, John L. Hopper, Ee Ming Wong, Melissa C. Southey

**Affiliations:** 1 Genetic Epidemiology Laboratory, Department of Pathology, The University of Melbourne, Parkville, VIC, 3010, Australia; 2 Colorectal Oncogenomics Group, Genetic Epidemiology Laboratory, Department of Pathology, The University of Melbourne, Parkville, VIC, Australia; 3 Centre for Epidemiology and Biostatistics, Melbourne School of Population and Global Health, The University of Melbourne, Parkville, VIC, Australia; 4 Cancer Epidemiology Centre, Cancer Council Victoria, Melbourne, VIC, Australia; Ohio State University Wexner Medical Center, UNITED STATES

## Abstract

DNA methylation can mimic the effects of both germline and somatic mutations for cancer predisposition genes such as *BRCA1* and *p16*^INK4a^. Constitutional DNA methylation of the *BRCA1* promoter has been well described and is associated with an increased risk of early-onset breast cancers that have *BRCA1*-mutation associated histological features. The role of methylation in the context of other breast cancer predisposition genes has been less well studied and often with conflicting or ambiguous outcomes. We examined the role of methylation in known breast cancer susceptibility genes in breast cancer predisposition and tumor development. We applied the Infinium HumanMethylation450 Beadchip (HM450K) array to blood and tumor-derived DNA from 43 women diagnosed with breast cancer before the age of 40 years and measured the methylation profiles across promoter regions of *BRCA1*, *BRCA2*, *ATM*, *PALB2*, *CDH1*, *TP53*, *FANCM*, *CHEK2*, *MLH1*, *MSH2*, *MSH6* and *PMS2*. Prior genetic testing had demonstrated that these women did not carry a germline mutation in *BRCA1*, *ATM*, *CHEK2*, *PALB2*, *TP53*, *BRCA2*, *CDH1 or FANCM*. In addition to the *BRCA1* promoter region, this work identified regions with variable methylation at multiple breast cancer susceptibility genes including *PALB2* and *MLH1*. Methylation at the region of *MLH1* in these breast cancers was not associated with microsatellite instability. This work informs future studies of the role of methylation in breast cancer susceptibility gene silencing.

## Introduction

Aberrant methylation patterns are a well-recognized feature of tumor cells and tumorigenic pathways. This includes genomic instability induced by global hypomethylation and silencing of several tumor suppressor genes via promoter methylation, which can act in a similar manner as do germline and somatic genetic mutations. [1].

Rare germline mutations in multiple genes (*BRCA1*, *BRCA2*, *ATM*, *PALB2*, *CDH1*, *TP53*, *FANCM*, *CHEK2*, *MLH1*, *MSH2*, *MSH6* and *PMS2*) are known to be associated with increased breast and/or ovarian cancer susceptibility and these genes are included in most commercial gene panel tests for breast and ovarian cancer susceptibility [2]. Research investigating the role of methylation as an alternate silencing mechanism for these genes has been limited.

*BRCA1* promoter hypermethylation in blood and breast tumor-derived DNA has been reported in the literature. *BRCA1* promoter methylation in blood-derived DNA is associated with a 3.5-fold (95% CI, 1.4–10.5) increased risk for early-onset breast cancer with histological features commonly seen in tumors arising in women with germline *BRCA1* mutations [3]. The silencing of *BRCA1* via promoter methylation has also been observed in breast tumors, including those arising in women with increased constitutional DNA methylation at this region [3–6]. Methylation of other breast cancer predisposition genes has been less well studied and often with conflicting outcomes.

Flanagan *et al*. performed methylation microarray analyses of peripheral blood DNA across genes including *BRCA1*, *BRCA2*, *CHEK2*, *ATM*, *TP53*, *CDH1* and *MLH1* and demonstrated gene body hypermethylation of *ATM* was associated with a 3-fold increase risk of breast cancer (P = 0.0017) [7]. Another study reported DNA methylation aberrations at an intragenic region of *ATM* to be associated with increased risk of breast cancer (increased risk for women in the upper quartile, OR 1.89; 95% CI 1.36–2.64; P = 2x10^-4^) [8].

The promoter methylation of *PALB2* has been investigated in the context of breast and ovarian cancer. Potapova *et al* (2008) reported promoter methylation of *PALB2* in approximately 8% of breast and ovarian cancers (including those with *BRCA2* germline mutations), detected by methylation specific PCR and Sanger sequencing [9]. However, Mikeska *et al* (2013) found little evidence of *PALB2* methylation in high-grade serous ovarian cancer using a methylation-sensitive high-resolution melting assay, and Poumpouridou *et al* (2016) were unable to detect *PALB2* promoter methylation in a series of 91 breast cancers [10,11]. Studies of *BRCA2* and *CHEK2* have found no evidence for regulation or silencing via methylation [12,13].

The goal of this study was to examine if low levels of constitutional DNA methylation corresponding to high levels of tumor DNA methylation could be identified at breast cancer predisposition genes other than *BRCA1*. We included blood and tumor-derived DNA from young affected women participating in the Australian Breast Cancer Family Study (ABCFS), using the Infinium HumanMethylation450 (HM450K) beadchip assay.

## Material and Methods

### Study samples

Blood and corresponding tumor-derived DNA samples were prepared from 43 women diagnosed with breast cancer before the age of forty participating in a population-based case-control component of the Australian Breast Cancer Family Registry (ABCFR) [14–16]. These women had been previously screened for germline mutations in *BRCA1*, *ATM*, *CHEK2*, *PALB2*, *TP53*, *BRCA2*, *CDH1*, and *FANCM* [14,15,17–26]. A subset of women had also been assessed for methylation at the *BRCA1* promoter using a site-specific MethyLight assay [3]. Written informed consent was obtained from each participant of the Australian Breast Cancer Family Study. This study was approved by the Human Research Ethics Committee of the University of Melbourne (Project 0608818) and meets the principles of the Declaration of Helsinki.

### DNA extraction from Guthrie card archival blood spots

Blood-derived DNA was extracted from archival dried blood spots (prepared for all participants of the ABCFR) on Whatman filter paper (GE Healthcare, United Kingdom) as previously described [27]. DNA was extracted from 28 (3.2mm diameter) punches per individual using the QIAamp 96 DNA Blood Kit (Qiagen; Hilden, Germany), as per the manufacturer’s protocol, except the DNA was eluted three times in 50μl nuclease free water to obtain a final volume of 150μl.

### DNA extraction from FFPE tumor sections

A haematoxylin and eosin (H&E) stained slide, marked up by a pathologist, was used as a reference for each case. The identified tumor-enriched tissue was macrodissected from between two to ten Methyl Green stained sections per tumor using both a scalpel (Swann-Morton, Sheffield, England) and a 21-guage syringe needle (Terumo, Tokyo, Japan), as previously described [28]. DNA was extracted using the QIAamp DNA FFPE Tissue Kit as per the manufacturer’s protocol (Qiagen; Hilden, Germany), varied only by an extended tissue incubation time of 48 hours with 20μl of Proteinase K (20mg/ml) replenished at 0 and 24 hours. Extracted tumor-derived DNA was eluted twice in 15μl elution buffer to obtain a final volume of 30μl. DNA samples were stored at 4°C.

### HumanMethylation450 beadchip assay

The HumanMethylation450 beadchip assay was run as previously described [27]. Briefly, the blood and tumor-derived DNA underwent sodium bisulfite modification using the EZ DNA Methylation-Gold^™^ Kit (Zymo Research, CA, United States), as per the manufacturer’s protocol. Bisulfite converted tumor-derived DNA was “restored” using the Infinium FFPE QC and DNA Restoration Kit as per the manufacturer’s protocol (Illumina, CA, United States). Sodium bisulfite modification was assessed using an in-house developed bisulfite-specific quantitative-PCR, as described previously [3].

The HM450K assay was performed as per the manufacturer’s protocol (Illumina, CA, United States). The extension and staining steps were performed using the TECAN automated liquid handler (Männedorf, Switzerland). The beadchips were scanned using the Illumina iScan (Illumina, CA, United States).

### Data quality check and statistical analysis

Raw methylation data was imported into the R statistical environment and processed using the bioconductor package *minfi 3*.*2* [29]. The data were filtered and normalized according to the Illumina protocol, in which the raw fluorescence data was normalized using control probes and methylation values (β-values) were calculated. Additional normalization using Subset-quantile Within Array Normalization (SWAN) was performed to adjust for technical discrepancies between Type I and Type II probes [30]. Probes with a mean detection P-value > 0.05 were excluded from further analysis. Differences of methylation between the blood and tumor-derived DNA were identified by a regression analysis using the empirical Bayes methods using *limma* R package. Statistical significance was estimated by FDR (false discovery rate) adjusted P-value cut-off of 0.01, calculated using moderated t-statistics. Selected breast cancer susceptibility genes were analyzed, which involved plotting the beta-methylation values at probes located at CpGs across the length of the gene. These regions were defined using UCSC Genome Browser [31].

### Microsatellite instability analysis

Microsatellite Instability (MSI) was assessed for 35 pairs of matched blood and tumor-derived DNA using the Promega MSI Analysis System, Version 1.2 Kit, as per manufacturers protocol (Promega, WI, United States). Due to the highly degraded nature of the samples, 10ng of DNA was used in each reaction instead of the recommended 1-2ng, and the number of amplification cycles was increased to 30. The output data was analyzed using GeneMarker, Version 2.6.7 (SoftGenetics, PA, United States). Tumors were considered to have high levels of microsatellite instability (MSI-H) when ≥2 out of the 5 markers showed mononucleotide repeat instability, whereas tumors with <2 unstable markers were considered microsatellite stable (MSS).

## Results

We assessed DNA methylation of the tumor and blood-derived samples from 43 women with early-onset breast cancer, across the genomic regions containing the breast and ovarian cancer susceptibility genes *BRCA1*, *BRCA2*, *PALB2*, *TP53*, *ATM*, *CDH1*, *CHEK2*, *FANCM*, *MLH1*, *MSH2*, *MSH6*, and *PMS2*.

### Tissue specific methylation

**Fig 1:**
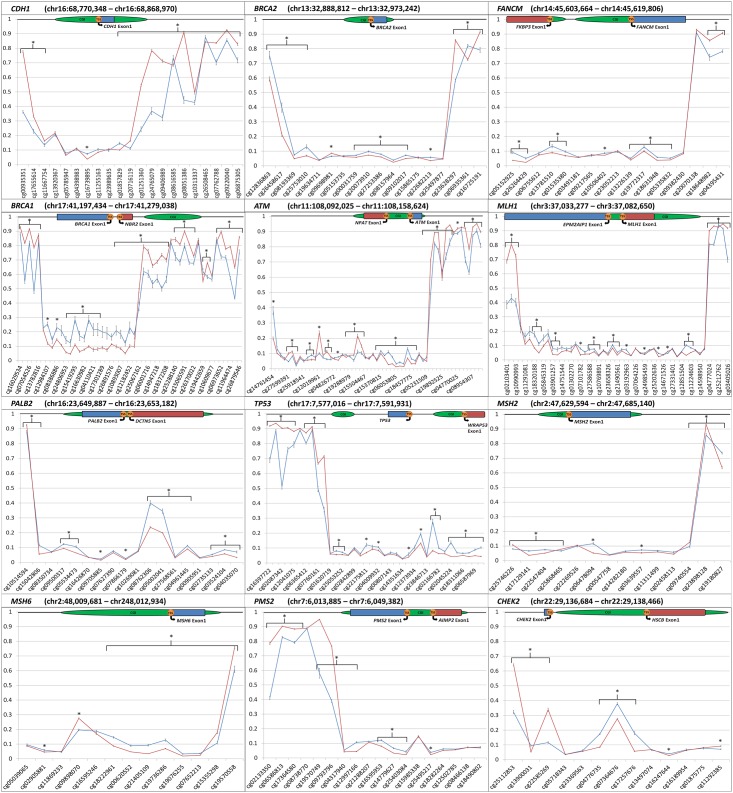
Comparison of average blood and tumor-derived DNA methylation across a panel of tumor suppressor genes. Plotted β-values at CpG probes across defined genomic regions. Red line indicates average β-value for blood-derived DNA samples. Blue line indicates average β-values for tumor-derived DNA samples. * indicates significant statistical difference (p<0.01). Error bars are plotted Standard Error of Mean. Gene region schematics denote [CGI (CpG Island), TSS (Transcriptional Start Site)].

The average beta-methylation values for blood and tumor-derived DNA at the promoter regions of the selected breast cancer susceptibility genes are shown in Fig 1. Tissue specific differences in average beta-methylation levels were identified at a number of CpG probes across each of the promoter regions as indicated.

As reported previously, increased tumor-derived DNA methylation was identified across all 15 *BRCA1* promoter-associated probes with an average Δβ of 11.24% (adj. P-value = 3x10^-15^ to 2x10^-3^) compared with blood-derived DNA (Fig 1) [Scott et al. manuscript under review]. Similarly, DNA methylation at 10 CpG probes across the *BRCA2* promoter region was increased in the tumor-derived DNA compared with blood-derived DNA (Δβ 5.49%; adj. P-value = 8x10^-9^ to 1x10^-5^). At *PALB2*, the tumor-derived DNA had elevated methylation across 12 promoter associated probes (Δβ 4.67%; adj. P-value = 3x10^-14^ to 1x10^-3^) particularly at two probes located 100bp from the Transcription Start Sites (TSS), cg08762306 (Δβ 16.30%; adj. P-value = 1x10^-11^) and cg05002041 (Δβ 14.59%; adj. P-value = 2x10^-11^). Methylation in the tumor-derived DNA was increased across 13 *TP53* promoter associated probes (Δβ 5.12%; adj. P-value = 5x10^-21^ to 1x10^-3^).

Increased methylation at 11 *ATM* promoter region probes proximal to the TSS in the tumor-derived DNA (Δβ 3.25%; adj. P-value = 1x10^-20^ to 9x10^-3^) was identified. Interestingly, this pattern of DNA methylation was reversed in nine gene body probes of *ATM* with an average Δβ of 12.80% (adj. P-value = 2x10^-28^ to 5x10^-5^). Overall, CpG probes encompassing *CDH1* measured similar methylation levels in the two tissue types across the CpG Island (CGI) and the first exon. However, methylation was significantly increased in the blood-derived DNA both upstream and downstream of this region, with an average Δβ of 21.26% across 12 probes (adj. P-value = 9x10^-33^ to 5x10^-4^). Three consecutive CpG probes in the *CHEK2* promoter region showed increased methylation (Δβ ~10.47%; adj. P-value = 2x10^-18^ to 5x10^-13^), and increases in methylation in the tumor-derived DNA were present consistently over nine *FANCM* promoter associated probes (Δβ 2.68%; adj. P-value = 3x10^-12^ to 3x10^-3^).

Small but significant increases (Δβ 3.49%; adj. P-value = 1x10^-22^ to 4x10^-3^) were observed over 14 probes at *MLH1* (located in close relation to the CGI), and four *MSH2* promoter associated probes (Δβ 3.46%; adj. P-value = 5x10^-15^ to 4x10^-6^). Tumor-derived DNA methylation at five probes across the gene body region of *PMS2* was significantly decreased (Δβ 25.70%; adj. P-value = 2x10^-32^ to 2x10^-16^), and increased methylation was measured at six promoter associated probes (Δβ 3.09%; adj. P-value = 9x10^-17^ to 6x10^-4^). CpG probes at *MSH6* showed consistently higher levels of methylation within the tumor-derived DNA across six probes, two of which were associated with its promoter (Δβ 4.09%; adj. P-value = 9x10^-17^ to 3x10^-5^) (Fig 1).

### Methylation in tumor-derived DNA samples

**Fig 2:**
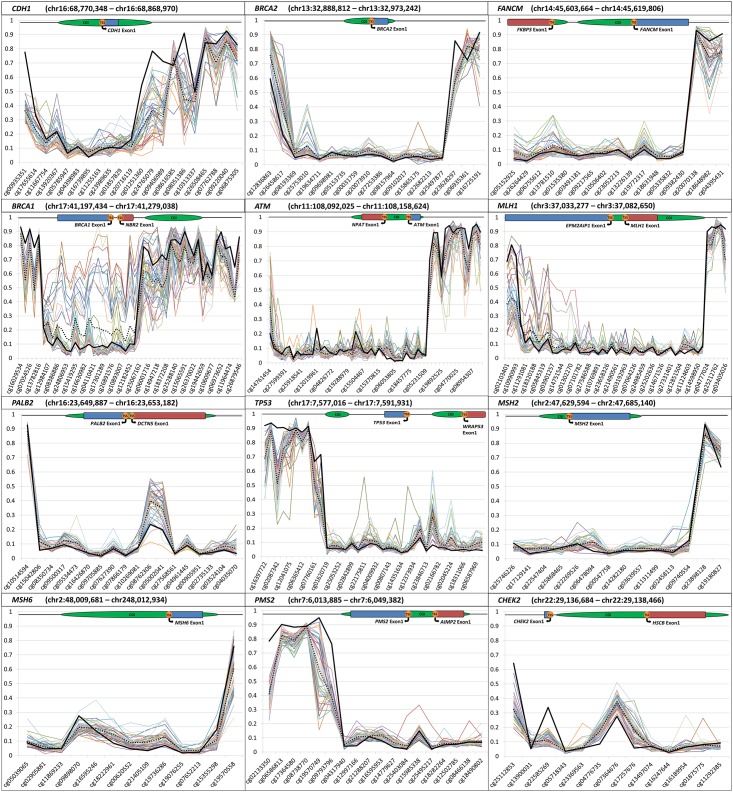
Sample tumor DNA methylation across a panel of tumor suppressor genes. Plotted β-values at CpG probes across defined genomic regions. Each colored line indicates β-values for an individual tumor-derived DNA sample. Solid black line indicates average β-values for blood-derived DNA. Dotted black line indicates average β-values for tumor-derived DNA. Gene region schematics denote [CGI (CpG Island), TSS (Transcriptional Start Site)].

We assessed the individual methylation pattern of each tumor-derived DNA sample across the genomic regions of interest (Fig 2).

As previously reported, 11 of the 43 samples had notably higher levels of methylation across 18 probes within the *BRCA1* promoter region. These 11 samples showed large increases in average methylation levels across the 18 probes when compared with the remaining 32 tumor-derived DNA samples (Δβ 25.03%), and a further increase when compared with the blood-derived DNA samples (Δβ 33.66%) (Scott et al. manuscript under review).

Extending the analysis to the other gene promoter regions of interest found that some tumor samples had distinct methylation profiles. For the *TP53* promoter region, one tumor showed notable increases in methylation across 13 probes when compared with the other tumor-derived DNAs (Δβ 14.65%). Two probes (cg22175811 and cg25896754), had a large average Δβ of 42.22%. In the *PMS2* promoter region, one sample had a large increase in methylation across 5 promoter-associated probes when compared with the remaining tumor-derived (Δβ ~14.66%) and blood-derived DNA samples (Δβ 15.44%). Both tumor samples with outlying methylation patterns at *TP53* and *PMS2* were infiltrating ductal carcinomas (grade III). Thirty seven of the 43 tumor samples (86%) were infiltrating ductal tumors.

For *PALB2*, 40 out of 43 most tumor-derived DNA samples had a higher level of methylation at two promoter probes (cg08762306 and cg05002041) when compared with the average blood-derived DNA methylation. However, one infiltrating ductal carcinoma had a large decrease in methylation compared with the other tumor (Δβ 37.88%) and blood-derived (Δβ 21.78%) DNA samples. Another high grade infiltrating ductal tumor-derived DNA sample had a consistent increase in methylation at *FANCM* across 12 consecutive probes when compared with the remaining 42 tumor samples (Δβ 8.99%). Two probes located in adjacent CGIs within the *FANCM* promoter region had a large degree of variation in tumor-derived DNA methylation. Methylation levels ranged from 34.12% to 5.09% at the probe upstream to the TSS (cg13781510), and 29.97% to 6.31% downstream to the TSS (cg19772317).

For *CDH1*, there was a large variation in methylation between the individual tumor-derived DNA samples, with levels ranging from 4.50% to 67.60% across three probes. *BRCA2* contained one promoter associated probe which also had large variation between the tumor-derived DNA samples, ranging from 4.49% to 41.88%.

For the *MSH6* promoter region, one tumor-derived DNA sample showed a consistently higher methylation level across 11 probes, located within a CGI and its north shore, when compared to the remaining 42 (Δβ ~8.37%). This sample also had increased methylation across the *BRCA1*-promoter region, and corresponded to an atypical medullary tumor. Interestingly, this sample also had a consistent increase in methylation across a number of other genes including *PALB2*, *TP53*, *CDH1*, *CHEK2*, *MSH2* and *FANCM*.

Two tumor-derived DNA samples were highly methylated at *MLH1* across 14 probes located proximal to a CGI nearby the promoter region. Methylation was greatly increased when compared to the other 41 tumor-derived DNA samples (average Δβ ~31.63%), in addition to the averaged blood-derived DNA values (Δβ ~35.46%). One sample was in infiltrating ductal tumor (grade II), whereas the other was an atypical medullary cancer (grade III) (Fig 2).

### MSI analysis

As *MLH1* promoter methylation has been shown to be associated with microsatellite instability (MSI), we tested the tumor derived DNA for evidence of these replication errors. MSI was examined for 35/43 (81%) paired blood and tumor-derived DNA samples, including those with increased *MLH1* and *MSH6* methylation, using five microsatellite markers: NR-21, NR-24, BAT-25, BAT-26, and MONO-27. No evidence of MSI was observed in any of the 35 samples tested (data not shown).

## Discussion

In this study, we assessed DNA methylation profiles of both blood and tumor-derived DNA from 43 early-onset breast cancer cases at known breast cancer predisposition genes.

**Fig 3:**
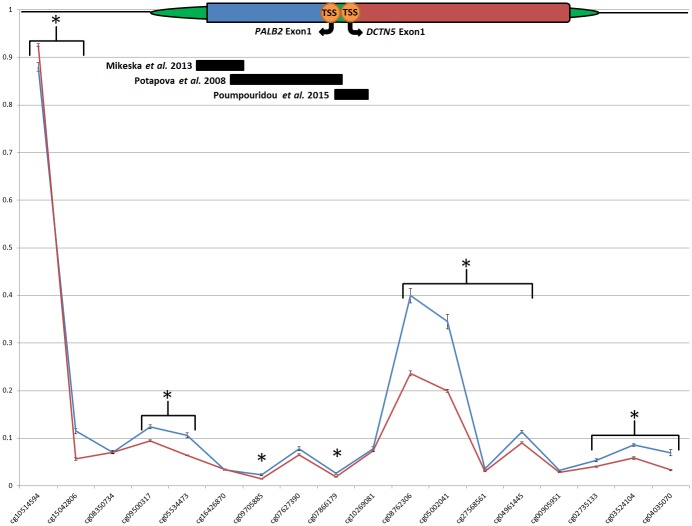
DNA methylation at the *PALB2* promoter region. Plotted β-values for each sample at each CpG probe across *PALB2* (chr16:23,649,887 –chr16:23,653,182). Red line indicates average β -value for blood-derived DNA samples. Blue line indicates average β-value for tumor-derived DNA samples. * indicates significant statistical difference (p<0.01). Solid black bars indicate regions previously screened for methylation. Gene region schematic denotes [CGI (CpG Island), TSS (Transcriptional Start Site)].

We found variable methylation in tumor-derived DNA at two *PALB2* promoter-associated CpG probes (cg08762306 and cg05002041). Three previous studies have investigated *PALB2* promoter methylation and each has targeted a different site within the CGI that encompasses the promoter region. Potapova *et al* (2008) found that 10/130 (~8%) sporadic breast and ovarian tumors presented with hypermethylation within the core promoter region of exon1 near the TSS [9]. Mikeska *et al* (2013) examined high-grade serous ovarian cancers and found none to be hypermethylated [10]. Poumpouridou *et al* (2016) analyzed 91 sporadic fresh-frozen breast tissues and also found none to be methylated and almost all (95.6%) to express *PALB2* [11]. The region this study found to be methylated was not targeted by any of the above studies (Fig 3). This new data should be used to inform the design of targeted methylation assessment of the *PALB2* promoter region in the future.

Studies of methylation and *TP53* in breast cancer are currently limited, although hypermethylation has been reported in epithelial ovarian and cervical cancers [32,33]. These two studies examined a region of TP53 that is encompassed by six HM450K CpG probes between chr17:7,590,728 and chr17:7,591,011. We observed no aberrant methylation in the tumor-derived DNA from this study at this region, however, marginal increased methylation was observed in the CpG probes 939bp downstream of this previously studied region (Fig 1). Increased methylation may be disrupting the normal mechanism of gene transcription, potentially contributing to tumorigenesis in these early-onset breast cancers. These CpGs may also vary in levels of methylation between different tumor tissue types.

**Fig 4:**
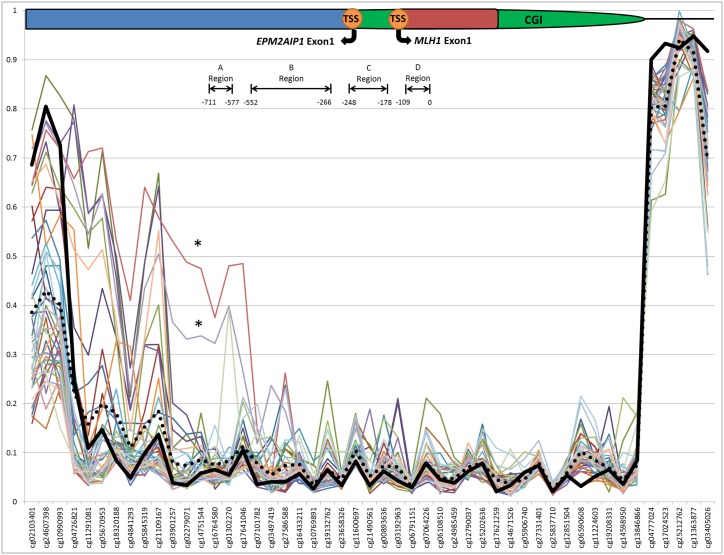
Sample tumor DNA methylation at the *MLH1* promoter region. Plotted β-values for each sample at each CpG probe across *MLH1* (chr3:37,033,277 –chr3:37,082,650). Solid black line indicates average β-values for blood-derived DNA. Dotted black line indicates average β-values for tumor-derived DNA. Schematic of four *MLH1* promoter regions previously defined by Deng *et al*, base pair number is in relation to the start codon (ATG) Regions. * highlights tumor-derived DNA samples showing increased *MLH1* methylation. Gene region schematic denotes [CGI (CpG Island), TSS (Transcriptional Start Site)]

Disruption to the normal function of the DNA mismatch repair (MMR) machinery causes MSI in ~15% of colorectal cancer cases, and has also been reported in sporadic breast tumors [34,35]. Loss of MMR function can result from either carrying a germline mutation in one of the MMR genes (Lynch syndrome), or by hypermethylation of the promoter region of *MLH1* in the tumor [36,37]. Two breast tumor-derived DNA samples from this study were highly methylated at *MLH1* across 14 probes when compared with the remaining 41 tumor-derived DNA samples (average Δβ ~31.63%). These 14 highly methylated probes were located across previously defined regions ‘A’ (-711 to -577) and ‘B’ (-552 to -266) upstream to the start codon of *MLH1* (chr3:37,033,779–37,034,673), although not within the ‘C’ region (-248 to -178) (Fig 4) [38]. Previous studies have found that methylation of the ‘C’ region of the promoter as opposed to the ‘A’ region is more critical for the transcriptional silencing of *MLH1*, and is associated with MSI-H status and loss of MLH1 protein expression in colorectal cancer [39]. Methylation of region ‘B’ may be important in silencing MLH1 expression, however further research is required to define the function of this region. In this study, increases in methylation were found within the ‘A’ and ‘B’ regions and therefore was consistent with our finding of absence of MSI-H in these two breast tumors.

Methylation at the *BRCA1* promoter in blood derived DNA is associated with risk of breast cancer with distinct histological features [3]. We tested for associations between methylation marks in the additional genes and histological features: mitotic index, nuclear grade, tubule formation, a trabecular growth pattern (primary or secondary), a syncytial growth pattern, pushing margins (>50%), circumscribed, necrosis, moderate or intense lymphocytic infiltrate but no clear associations were identified.

This study has limitations. First, the study is small and the molecular events being investigated could be very rare in breast cancer predisposition and tumorigenesis. Larger studies are needed to further test for i) methylation as a silencing mechanism involved with these genes and breast cancer predisposition, ii) methylation as a silencing mechanism involved with these genes and breast cancer progression and iii) for associations between methylation marks and histological features.

Second, HM450K array data derived from adjacent non-tumour ductal epithelium (preferably matched to the women whose tumors were included in this study), would have been an informative reference. Unfortunately, accessing normal breast material is a significant challenge, especially when using archival FFPE tumor material [40] and the necessary resources were not available for this study. However, as demonstrated by the *BRCA1* example (see Fig 2; *BRCA1*) much information is gained by tumor/tumor and tumor/blood comparisons and it is highly unlikely that a methylation event at one of these breast cancer predisposition genes would be involved in all tumor and blood derived DNA samples included in this study. Indeed, aberrant methylation at *BRCA1* is evident in only eleven tumor DNA samples (and the corresponding blood derived DNA samples) despite the highly selected nature of the samples (early onset breast cancer).

Third, although we have indirectly tested the relevance of the observed *MLH1* methylation (by testing for microsatellite instability), we do not have any direct evidence that the methylation patterns described for these breast cancer predisposition genes correspond to a change in gene or protein expression (due to a lack of suitable material from which to collect this data).

Application of the HM450K beadchip assay has enabled a finer description of DNA methylation at genomic regions of interest to breast cancer susceptibility and tumor progression. This work informs future studies investigating the role of methylation in breast cancer susceptibility gene silencing.

## Supporting Information

S1 Data Dataset. (CSV)
